# Bis(acetyl­acetonato-κ^2^ *O*,*O*′)(2-amino-1-methyl-1*H*-benzimidazole-κ*N* ^3^)oxido­vanadium(IV)

**DOI:** 10.1107/S1600536809023113

**Published:** 2009-06-24

**Authors:** Zukhra Ch. Kadirova, Dilnoza S. Rahmonova, Samat A. Talipov, Jamshid M. Ashurov, Nusrat A. Parpiev

**Affiliations:** a National University of Uzbekistan, Tashkent 100123, Uzbekistan; b Institute of Biorganic Chemistry, Mirzo-Ulugbek St. 83, Tashkent 100125, Uzbekistan

## Abstract

The title mixed-ligand oxidovanadium(IV) compound, [VO(C_5_H_7_O_2_)_2_(C_8_H_9_N_3_)], contains a V^IV^ atom in a distorted octahedral coordination, which is typical for such complexes. The vanadyl group and the *N*-heterocyclic ligand are *cis* to each other. The coordination bond is located at the endocyclic N atom of the benzimidazole ligand. Intra­molecular hydrogen bonds between the *exo*-NH_2_ group H atoms and acetyl­acetonate O atoms stabilize the crystal structure.

## Related literature

For the activity of vanadium complexes, see: Rehder (1999 ▶). For the crystal structures of acetylacetonate and benzimidazole oxidovanadium(IV) and (V) complexes, see: Maurya (2002 ▶); Caira *et al.* (1972 ▶); Shao *et al.* (1984 ▶); Crans *et al.* (1997 ▶); Maurya *et al.* (2006 ▶); Akhmed *et al.* (2004 ▶).  For 1-methyl- 2-aminobenzimidazole compounds, see: Borodkina *et al.* (2003 ▶); Chekhlov (2004 ▶).
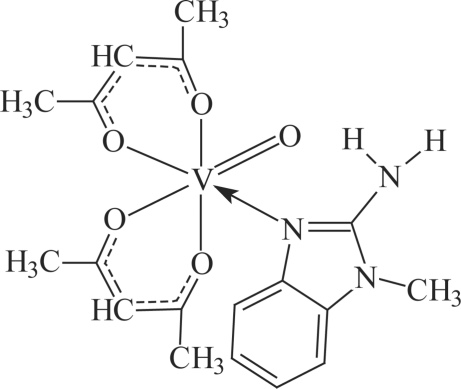


## Experimental

### 

#### Crystal data

[V(C_5_H_7_O_2_)_2_O(C_8_H_9_N_3_)]
*M* *_r_* = 412.33
Monoclinic, 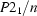

*a* = 8.27120 (10) Å
*b* = 15.0472 (2) Å
*c* = 16.1078 (2) Å
β = 104.2646 (14)°
*V* = 1942.94 (4) Å^3^
*Z* = 4
Cu *K*α radiation
μ = 4.57 mm^−1^
*T* = 293 K
0.25 × 0.12 × 0.08 mm

#### Data collection

Oxford Diffraction Xcalibur diffractometer
Absorption correction: multi-scan (*CrysAlisPro*; Oxford Diffraction, 2007 ▶) *T*_min_ = 0.544, *T*_max_ = 0.694
8892 measured reflections
3720 independent reflections
2983 reflections with *I* > 2σ(*I*)
*R*_int_ = 0.028

#### Refinement

*R*[*F* ^2^ > 2σ(*F* ^2^)] = 0.037
*wR*(*F* ^2^) = 0.106
*S* = 1.00
3720 reflections
249 parameters
H-atom parameters constrained
Δρ_max_ = 0.20 e Å^−3^
Δρ_min_ = −0.23 e Å^−3^

### 

Data collection: *CrysAlisPro* (Oxford Diffraction, 2007 ▶); cell refinement: *CrysAlisPro*; data reduction: *CrysAlisPro*; program(s) used to solve structure: *SHELXS97* (Sheldrick, 2008 ▶); program(s) used to refine structure: *SHELXL97* (Sheldrick, 2008 ▶); molecular graphics: *ORTEP-3 for Windows* (Farrugia, 1997 ▶); software used to prepare material for publication: *SHELXTL* (Sheldrick, 2008 ▶).

## Supplementary Material

Crystal structure: contains datablocks I, global. DOI: 10.1107/S1600536809023113/su2113sup1.cif

Structure factors: contains datablocks I. DOI: 10.1107/S1600536809023113/su2113Isup2.hkl

Additional supplementary materials:  crystallographic information; 3D view; checkCIF report

## Figures and Tables

**Table 1:** Hydrogen-bond geometry (Å, °)

*D*—H⋯*A*	*D*—H	H⋯*A*	*D*⋯*A*	*D*—H⋯*A*
N3*A*—H3*AA*⋯O2*B*	0.86	2.38	2.972 (3)	127
N3*A*—H3*AA*⋯O2*C*	0.86	2.47	3.034 (2)	124

**Fig. 1.**
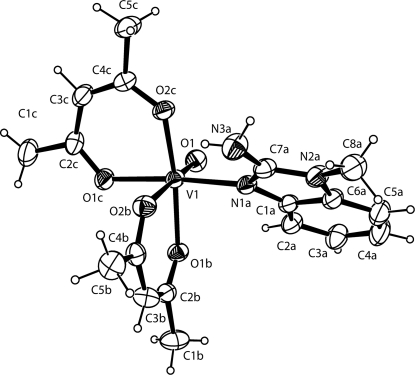
A view of the molecuar structure of the title compound, showing the atom numbering scheme. Displacement ellipsoids are drawn at the 30% probability level.

**Fig. 2.**
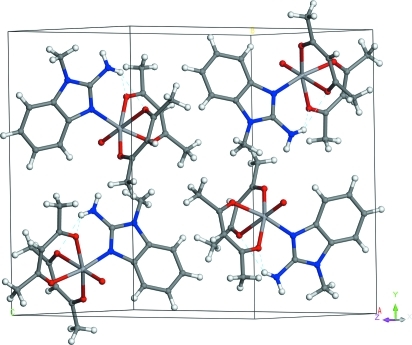
A view of the crystal packing of the title compound, with the intramolecuéar N—H···O hydrogen bonds shown as pale blue dashed lines (see Table 1 for details).

## supplementary crystallographic information

### Comment

Vanadium complexes have attracted interest in recent nears due to their insulin-mimetic action, and to their activity in nitrogen fixation and haloperoxidation (Rehder, 1999). The vanadium atom can have different coordination numbers and forms coordination compounds with a variety of coordination geometries and oxidation states (Maurya, 2002). Limited information is available on the crystal structure of the vanadium complexes of substituted benzimidazoles (Crans *et al.*, 1997; Maurya *et al.*, 2006). Bis(acetylacetonato)oxovanadium, [VO(acac)_2_], is a common precursor for the synthesis of the mixed ligand vanadium(IV) and vanadium(V) complexes with the N-containing monodentate ligands (*L*), [VO(acac)_2_L]. Usually these bis-chelated complexes can be *cis-* or *trans-* with distorted octahedral configurations (Caira *et al.*, 1972; Shao *et al.*, 1984). In this study, we prepared the mixed-ligand complex of oxovanadium(IV) with bidentate acetylacetonate and the monodentate benzimidazole, 2-amino-1-methylbenzimidazole, and report herein on its crystal structure.

The molecular structure of the title compound is shown in Fig. 1, and geometrical parameters are available from the archived CIF. In this *cis*-complex, [VO(acac)_2_L], the metal center has a slightly distorted octahedral N_1_O_5_ coordination sphere, assembled by the O—O-donor acetylacetonate, the oxo-group and the pyridine N-atom of the benzimidazole. The angles around the vanadium atom deviate from 90°, being in the range of 80.85 (6) - 99.91 (7) °, and from 180°, being in the range of 164.96 (7) - 179.18 (7)°, due to coordination of the sterically large ligand to the five-coordinate square-pyramidal [VO(acac)_2_] complex (Akhmed *et al.*, 2004).

The coordination bond is localized at the *endo*-cyclic N-atom of the benzimidazole ligand and the bond lengths and angles are similar to those reported for 2-amino-1-methylbenzimidazolium chloride hydrate (Borodkina *et al.*, 2003), and bis(2-amino-1-methylbenzimidazole-N) dichlorocobalt(II) (Chekhlov *et al.*, 2004). The amino-group is coplanar with the methyl-group [torsion angle C8A—N2A—C7A—N3A is 4.3 (4)°] and participates in intramolecular hydrogen bonds with the carbonyl O-atoms (Fig.2 and Table 1).

The V—O bond (V1—O2B) *trans* to the *oxo*-group is significally longer (2.1523 (17) Å) than the V—O bonds which are *cis* to the oxo-group (1.9927 (14) - 2.0139 (14) Å). In contrast the carbonyl bond involving atom O2B (C4B?O2B) is shorter, (1.252 (3) Å), than the other acetylacetonate C?O bonds [1.264 (3) - 1.273 (3) Å]. The V—N bond length, the *cis-* and *trans-* V—O bond lengths are comparable to those reported for oxovanadium(IV) species containing acac^-^ as ligand in a similar orientation (Crans *et al.*, 1997).

### Experimental

Equimolar quantities of [VO(acac)_2_] (acac = acetnlacetonate) and 2-amine-1-methylbenzimidazole (0.53 g, 1.9 mmol) were refluxed in ethanol for 3 h. The resulting green solution yielded green crystals which were filtered off and washed twice with acetone. Elem. Analysis found: C 52.4, H 6.0, N 10.3, V 12.4%; C_18_H_23_N_3_O_5_V requires: C 52.4, H 5.6, N 10.2,V 12.4%. IR (BRUKER spectrometer, KBr, cm^-1^): 3415 s, 3326 s, 1641 s, 1591 s, 1556 s, 1462*m*, 1373 s, 1273*m*, 1018*m*, 1252w, 1198w, 1132w,1052*m*, 983*m*, 939*m*, 787w, 746*m*, 669w, 590w, 557w, 455w). Crystals of the title compound, suitable X-ray diffraction analysis, were selected directly from the sample as prepared.

### Refinement

All the H-atoms were included in calculated positions [N—H = 0.88 Å, C—H = 0.93 - 0.96 Å] and treated as riding atoms [*U*_iso_(H) = k × *U*_eq_(parent atom], where k = 1.2 for NH_2_ and CH H atoms and 1.5 for methyl H atoms].

### Crystal data

**Table:**

[V(C_5_H_7_O_2_)_2_O(C_8_H_9_N_3_)]	*F*(000) = 860
*M**_r_* = 412.33	*D*_x_ = 1.410 Mg m^−^^3^
Monoclinic, *P*2_1_/*n*	Cu *K*α radiation, λ = 1.54184 Å
Hall symbol: -P 2yn	Cell parameters from 3720 reflections
*a* = 8.2712 (1) Å	θ = 4.1–76.0°
*b* = 15.0472 (2) Å	µ = 4.57 mm^−^^1^
*c* = 16.1078 (2) Å	*T* = 293 K
β = 104.2646 (14)°	Monoclinic, green
*V* = 1942.94 (4) Å^3^	0.25 × 0.12 × 0.08 mm
*Z* = 4	

### Data collection

**Table:**

Oxford Diffraction Xcalibur diffractometer	2983 reflections with *I* > 2σ(*I*)
Radiation source: fine-focus sealed tube	*R*_int_ = 0.028
graphite	θ_max_ = 76.0°, θ_min_ = 4.1°
heavy atom scans	*h* = −9→9
Absorption correction: multi-scan (*CrysAlis PRO*; Oxford Diffraction, 2007)	*k* = −18→17
*T*_min_ = 0.544, *T*_max_ = 0.694	*l* = −20→20
8892 measured reflections	3 standard reflections every 120 reflections
3720 independent reflections	intensity decay: none

### Refinement

**Table:**

Refinement on *F*^2^	Primary atom site location: structure-invariant direct methods
Least-squares matrix: full	Secondary atom site location: difference Fourier map
*R*[*F*^2^ > 2σ(*F*^2^)] = 0.037	Hydrogen site location: inferred from neighbouring sites
*wR*(*F*^2^) = 0.106	H-atom parameters constrained
*S* = 1.00	*w* = 1/[σ^2^(*F*_o_^2^) + (0.0695*P*)^2^] where *P* = (*F*_o_^2^ + 2*F*_c_^2^)/3
3720 reflections	(Δ/σ)_max_ = 0.001
249 parameters	Δρ_max_ = 0.20 e Å^−^^3^
0 restraints	Δρ_min_ = −0.23 e Å^−^^3^

### Special details

**Table:**

Geometry. Bond distances, angles *etc*. have been calculated using the rounded fractional coordinates. All su's are estimated from the variances of the (full) variance-covariance matrix. The cell e.s.d.'s are taken into account in the estimation of distances, angles and torsion angles
Refinement. Refinement of *F*^2^ against ALL reflections. The weighted *R*-factor *wR* and goodness of fit *S* are based on *F*^2^, conventional *R*-factors *R* are based on *F*, with *F* set to zero for negative *F*^2^. The threshold expression of *F*^2^ > σ(*F*^2^) is used only for calculating *R*-factors(gt) *etc*. and is not relevant to the choice of reflections for refinement. *R*-factors based on *F*^2^ are statistically about twice as large as those based on *F*, and *R*- factors based on ALL data will be even larger.

### Fractional atomic coordinates and isotropic or equivalent isotropic displacement parameters (Å^2^)

**Table:**

	*x*	*y*	*z*	*U*_iso_*/*U*_eq_	
V1	0.72939 (4)	0.84139 (2)	0.15879 (2)	0.0439 (1)	
O1B	0.85987 (19)	0.94296 (8)	0.22441 (9)	0.0495 (5)	
O1C	0.7792 (2)	0.89026 (9)	0.05202 (9)	0.0572 (5)	
O1V	0.5517 (2)	0.88731 (10)	0.14793 (10)	0.0590 (5)	
O2B	0.9707 (2)	0.78057 (10)	0.17502 (10)	0.0556 (5)	
O2C	0.6598 (2)	0.72797 (9)	0.09633 (9)	0.0559 (5)	
N1A	0.7312 (2)	0.77605 (10)	0.27641 (10)	0.0450 (5)	
N2A	0.7430 (3)	0.67520 (11)	0.37942 (11)	0.0579 (7)	
N3A	0.8363 (3)	0.63142 (12)	0.25875 (13)	0.0667 (8)	
C1A	0.6736 (3)	0.81453 (13)	0.34241 (12)	0.0475 (6)	
C1B	1.0649 (4)	1.03962 (17)	0.3018 (2)	0.0777 (10)	
C1C	0.7707 (5)	0.9161 (2)	−0.09262 (17)	0.0869 (13)	
C2A	0.6149 (3)	0.89931 (15)	0.35119 (14)	0.0577 (8)	
C2B	1.0157 (3)	0.95299 (13)	0.25591 (12)	0.0497 (7)	
C2C	0.7364 (3)	0.85815 (15)	−0.02274 (15)	0.0574 (8)	
C3A	0.5629 (4)	0.91763 (18)	0.42447 (17)	0.0732 (10)	
C3B	1.1380 (3)	0.89314 (15)	0.25154 (17)	0.0630 (8)	
C3C	0.6646 (4)	0.77473 (16)	−0.04198 (15)	0.0637 (8)	
C4A	0.5667 (5)	0.8537 (2)	0.48746 (18)	0.0844 (13)	
C4B	1.1117 (3)	0.80980 (14)	0.21204 (13)	0.0505 (7)	
C4C	0.6354 (3)	0.71403 (14)	0.01637 (14)	0.0523 (7)	
C5A	0.6251 (4)	0.76985 (18)	0.47967 (16)	0.0768 (12)	
C5B	1.2601 (3)	0.75171 (16)	0.21416 (18)	0.0698 (9)	
C5C	0.5724 (4)	0.62284 (17)	−0.01264 (17)	0.0725 (9)	
C6A	0.6788 (3)	0.75212 (14)	0.40690 (13)	0.0562 (7)	
C7A	0.7706 (3)	0.69247 (13)	0.30215 (13)	0.0504 (7)	
C8A	0.7863 (4)	0.59453 (16)	0.43059 (17)	0.0768 (9)	
H3AA	0.85610	0.64500	0.21040	0.0800*	
H3AB	0.85830	0.57890	0.27940	0.0800*	
H2AA	0.61060	0.94230	0.30920	0.0690*	
H3AC	0.52420	0.97440	0.43200	0.0880*	
H4A	0.52880	0.86830	0.53550	0.1020*	
H5AA	0.62850	0.72680	0.52150	0.0920*	
H8AA	0.86740	0.56120	0.41000	0.1150*	
H8AB	0.68810	0.55900	0.42600	0.1150*	
H8AC	0.83190	0.61030	0.48950	0.1150*	
H1BA	1.04180	1.08760	0.26140	0.1170*	
H1BB	1.18190	1.03870	0.32930	0.1170*	
H1BC	1.00230	1.04780	0.34420	0.1170*	
H3BA	1.24760	0.90920	0.27690	0.0750*	
H5BA	1.22290	0.69420	0.19160	0.1050*	
H5BB	1.32470	0.74570	0.27220	0.1050*	
H5BC	1.32750	0.77810	0.18010	0.1050*	
H1CA	0.87820	0.94350	−0.07300	0.1300*	
H1CB	0.68650	0.96130	−0.10720	0.1300*	
H1CC	0.76940	0.88050	−0.14220	0.1300*	
H3CA	0.63350	0.75860	−0.09950	0.0760*	
H5CA	0.65170	0.57920	0.01560	0.1090*	
H5CB	0.55760	0.61810	−0.07350	0.1090*	
H5CC	0.46750	0.61290	0.00140	0.1090*	

### Atomic displacement parameters (Å^2^)

**Table:**

	*U*^11^	*U*^22^	*U*^33^	*U*^12^	*U*^13^	*U*^23^
V1	0.0518 (2)	0.0385 (2)	0.0396 (2)	−0.0028 (1)	0.0080 (1)	−0.0025 (1)
O1B	0.0574 (9)	0.0391 (7)	0.0499 (8)	−0.0030 (6)	0.0091 (6)	−0.0053 (6)
O1C	0.0787 (11)	0.0472 (8)	0.0483 (8)	−0.0081 (7)	0.0207 (7)	−0.0005 (6)
O1V	0.0571 (10)	0.0618 (9)	0.0548 (9)	0.0055 (7)	0.0073 (7)	0.0089 (7)
O2B	0.0558 (10)	0.0477 (8)	0.0632 (9)	0.0019 (6)	0.0144 (7)	−0.0092 (6)
O2C	0.0729 (11)	0.0472 (8)	0.0433 (7)	−0.0124 (7)	0.0063 (7)	−0.0051 (6)
N1A	0.0543 (10)	0.0383 (8)	0.0401 (8)	−0.0030 (7)	0.0071 (7)	−0.0021 (6)
N2A	0.0848 (15)	0.0398 (9)	0.0435 (9)	−0.0063 (9)	0.0054 (9)	0.0015 (7)
N3A	0.0966 (17)	0.0426 (9)	0.0608 (12)	0.0106 (10)	0.0193 (11)	−0.0009 (8)
C1A	0.0541 (13)	0.0442 (10)	0.0409 (10)	−0.0077 (9)	0.0053 (8)	−0.0041 (8)
C1B	0.084 (2)	0.0505 (13)	0.0874 (19)	−0.0150 (12)	0.0000 (15)	−0.0131 (12)
C1C	0.130 (3)	0.0822 (18)	0.0595 (15)	−0.0136 (18)	0.0444 (16)	−0.0004 (13)
C2A	0.0689 (15)	0.0514 (12)	0.0503 (12)	0.0024 (10)	0.0097 (10)	−0.0021 (9)
C2B	0.0621 (14)	0.0430 (10)	0.0415 (10)	−0.0093 (9)	0.0083 (9)	0.0022 (8)
C2C	0.0703 (16)	0.0576 (13)	0.0494 (11)	0.0073 (11)	0.0246 (10)	−0.0027 (9)
C3A	0.097 (2)	0.0639 (15)	0.0623 (14)	0.0086 (14)	0.0263 (14)	−0.0116 (12)
C3B	0.0499 (14)	0.0591 (13)	0.0736 (15)	−0.0065 (10)	0.0033 (11)	−0.0042 (11)
C3C	0.0863 (18)	0.0616 (14)	0.0451 (11)	−0.0049 (12)	0.0199 (11)	−0.0113 (10)
C4A	0.121 (3)	0.087 (2)	0.0534 (14)	−0.0039 (17)	0.0369 (16)	−0.0130 (13)
C4B	0.0537 (13)	0.0511 (11)	0.0477 (10)	0.0020 (9)	0.0146 (9)	0.0085 (8)
C4C	0.0554 (13)	0.0492 (11)	0.0489 (11)	0.0013 (9)	0.0064 (9)	−0.0105 (9)
C5A	0.118 (3)	0.0664 (16)	0.0471 (12)	−0.0123 (15)	0.0222 (14)	0.0000 (11)
C5B	0.0621 (17)	0.0719 (16)	0.0744 (16)	0.0144 (12)	0.0150 (13)	0.0031 (12)
C5C	0.095 (2)	0.0597 (14)	0.0575 (14)	−0.0135 (13)	0.0088 (13)	−0.0165 (11)
C6A	0.0747 (16)	0.0492 (11)	0.0415 (10)	−0.0104 (10)	0.0083 (10)	−0.0029 (8)
C7A	0.0606 (14)	0.0395 (10)	0.0462 (10)	−0.0023 (9)	0.0038 (9)	−0.0032 (8)
C8A	0.115 (2)	0.0467 (12)	0.0609 (14)	−0.0096 (13)	0.0069 (15)	0.0115 (10)

### Geometric parameters (Å, °)

**Table:**

V1—O1B	2.0139 (14)	C3C—C4C	1.374 (3)
V1—O1C	2.0044 (15)	C4A—C5A	1.368 (4)
V1—O1V	1.5942 (17)	C4B—C5B	1.500 (3)
V1—O2B	2.1523 (17)	C4C—C5C	1.501 (3)
V1—O2C	1.9927 (14)	C5A—C6A	1.378 (4)
V1—N1A	2.1313 (16)	C1B—H1BA	0.9600
O1B—C2B	1.273 (3)	C1B—H1BB	0.9600
O1C—C2C	1.264 (3)	C1B—H1BC	0.9600
O2B—C4B	1.252 (3)	C1C—H1CA	0.9600
O2C—C4C	1.271 (3)	C1C—H1CB	0.9600
N1A—C1A	1.394 (3)	C1C—H1CC	0.9600
N1A—C7A	1.339 (2)	C2A—H2AA	0.9300
N2A—C6A	1.391 (3)	C3A—H3AC	0.9300
N2A—C7A	1.345 (3)	C3B—H3BA	0.9300
N2A—C8A	1.461 (3)	C3C—H3CA	0.9300
N3A—C7A	1.347 (3)	C4A—H4A	0.9300
N3A—H3AB	0.8600	C5A—H5AA	0.9300
N3A—H3AA	0.8600	C5B—H5BA	0.9600
C1A—C2A	1.385 (3)	C5B—H5BB	0.9600
C1A—C6A	1.393 (3)	C5B—H5BC	0.9600
C1B—C2B	1.504 (3)	C5C—H5CA	0.9600
C1C—C2C	1.505 (4)	C5C—H5CB	0.9600
C2A—C3A	1.380 (4)	C5C—H5CC	0.9600
C2B—C3B	1.369 (3)	C8A—H8AA	0.9600
C2C—C3C	1.391 (3)	C8A—H8AB	0.9600
C3A—C4A	1.393 (4)	C8A—H8AC	0.9600
C3B—C4B	1.399 (3)		
			
O1B—V1—O1C	88.57 (6)	C1A—C6A—C5A	123.1 (2)
O1B—V1—O1V	95.11 (7)	N2A—C6A—C1A	105.53 (18)
O1B—V1—O2B	84.14 (6)	N2A—C6A—C5A	131.3 (2)
O1B—V1—O2C	164.96 (7)	N1A—C7A—N2A	112.59 (18)
O1B—V1—N1A	89.89 (6)	N1A—C7A—N3A	125.33 (19)
O1C—V1—O1V	97.22 (7)	N2A—C7A—N3A	122.05 (19)
O1C—V1—O2B	83.09 (6)	C2B—C1B—H1BA	110.00
O1C—V1—O2C	88.62 (6)	C2B—C1B—H1BB	110.00
O1C—V1—N1A	166.93 (7)	C2B—C1B—H1BC	109.00
O1V—V1—O2B	179.18 (7)	H1BA—C1B—H1BB	109.00
O1V—V1—O2C	99.91 (7)	H1BA—C1B—H1BC	109.00
O1V—V1—N1A	95.85 (7)	H1BB—C1B—H1BC	109.00
O2B—V1—O2C	80.85 (6)	C2C—C1C—H1CA	109.00
O2B—V1—N1A	83.84 (6)	C2C—C1C—H1CB	110.00
O2C—V1—N1A	89.52 (6)	C2C—C1C—H1CC	109.00
V1—O1B—C2B	131.18 (13)	H1CA—C1C—H1CB	109.00
V1—O1C—C2C	127.48 (15)	H1CA—C1C—H1CC	109.00
V1—O2B—C4B	129.48 (14)	H1CB—C1C—H1CC	109.00
V1—O2C—C4C	127.37 (13)	C1A—C2A—H2AA	121.00
V1—N1A—C1A	123.89 (12)	C3A—C2A—H2AA	121.00
V1—N1A—C7A	131.02 (14)	C2A—C3A—H3AC	119.00
C1A—N1A—C7A	104.89 (16)	C4A—C3A—H3AC	119.00
C6A—N2A—C7A	107.40 (17)	C2B—C3B—H3BA	117.00
C6A—N2A—C8A	124.86 (19)	C4B—C3B—H3BA	117.00
C7A—N2A—C8A	127.5 (2)	C2C—C3C—H3CA	117.00
H3AA—N3A—H3AB	120.00	C4C—C3C—H3CA	117.00
C7A—N3A—H3AA	120.00	C3A—C4A—H4A	119.00
C7A—N3A—H3AB	120.00	C5A—C4A—H4A	120.00
N1A—C1A—C6A	109.59 (17)	C4A—C5A—H5AA	122.00
N1A—C1A—C2A	130.93 (18)	C6A—C5A—H5AA	122.00
C2A—C1A—C6A	119.5 (2)	C4B—C5B—H5BA	109.00
C1A—C2A—C3A	117.6 (2)	C4B—C5B—H5BB	109.00
O1B—C2B—C1B	115.1 (2)	C4B—C5B—H5BC	109.00
O1B—C2B—C3B	126.17 (19)	H5BA—C5B—H5BB	109.00
C1B—C2B—C3B	118.8 (2)	H5BA—C5B—H5BC	110.00
O1C—C2C—C3C	124.1 (2)	H5BB—C5B—H5BC	109.00
O1C—C2C—C1C	115.5 (2)	C4C—C5C—H5CA	109.00
C1C—C2C—C3C	120.3 (2)	C4C—C5C—H5CB	109.00
C2A—C3A—C4A	122.0 (3)	C4C—C5C—H5CC	109.00
C2B—C3B—C4B	125.4 (2)	H5CA—C5C—H5CB	109.00
C2C—C3C—C4C	125.8 (2)	H5CA—C5C—H5CC	109.00
C3A—C4A—C5A	121.0 (3)	H5CB—C5C—H5CC	109.00
C3B—C4B—C5B	118.5 (2)	N2A—C8A—H8AA	110.00
O2B—C4B—C5B	117.85 (19)	N2A—C8A—H8AB	110.00
O2B—C4B—C3B	123.6 (2)	N2A—C8A—H8AC	110.00
O2C—C4C—C3C	124.9 (2)	H8AA—C8A—H8AB	109.00
O2C—C4C—C5C	115.0 (2)	H8AA—C8A—H8AC	109.00
C3C—C4C—C5C	120.1 (2)	H8AB—C8A—H8AC	109.00
C4A—C5A—C6A	116.9 (2)		
			
O1C—V1—O1B—C2B	−83.35 (17)	V1—N1A—C1A—C2A	−4.2 (3)
O1V—V1—O1B—C2B	179.53 (17)	C1A—N1A—C7A—N3A	−178.0 (2)
O2B—V1—O1B—C2B	−0.15 (17)	V1—N1A—C7A—N3A	7.1 (4)
N1A—V1—O1B—C2B	83.67 (17)	C7A—N1A—C1A—C6A	−0.4 (3)
O1B—V1—O1C—C2C	178.64 (19)	C1A—N1A—C7A—N2A	−0.2 (3)
O1V—V1—O1C—C2C	−86.4 (2)	C7A—N1A—C1A—C2A	−179.6 (3)
O2B—V1—O1C—C2C	94.37 (19)	C8A—N2A—C6A—C5A	−7.5 (5)
O2C—V1—O1C—C2C	13.42 (19)	C8A—N2A—C7A—N1A	−173.6 (2)
O1B—V1—O2B—C4B	0.1 (2)	C7A—N2A—C6A—C5A	177.9 (3)
O1C—V1—O2B—C4B	89.40 (18)	C8A—N2A—C7A—N3A	4.3 (4)
O2C—V1—O2B—C4B	179.11 (19)	C6A—N2A—C7A—N1A	0.8 (3)
N1A—V1—O2B—C4B	−90.39 (18)	C6A—N2A—C7A—N3A	178.6 (2)
O1C—V1—O2C—C4C	−11.2 (2)	C7A—N2A—C6A—C1A	−1.0 (3)
O1V—V1—O2C—C4C	85.9 (2)	C8A—N2A—C6A—C1A	173.6 (2)
O2B—V1—O2C—C4C	−94.4 (2)	N1A—C1A—C2A—C3A	178.8 (3)
N1A—V1—O2C—C4C	−178.3 (2)	N1A—C1A—C6A—C5A	−178.1 (2)
O1B—V1—N1A—C1A	55.10 (17)	N1A—C1A—C6A—N2A	0.8 (3)
O1B—V1—N1A—C7A	−130.9 (2)	C2A—C1A—C6A—N2A	−179.9 (2)
O1V—V1—N1A—C1A	−40.02 (17)	C2A—C1A—C6A—C5A	1.2 (4)
O1V—V1—N1A—C7A	134.0 (2)	C6A—C1A—C2A—C3A	−0.4 (4)
O2B—V1—N1A—C1A	139.22 (17)	C1A—C2A—C3A—C4A	−0.7 (4)
O2B—V1—N1A—C7A	−46.7 (2)	C1B—C2B—C3B—C4B	179.3 (2)
O2C—V1—N1A—C1A	−139.93 (17)	O1B—C2B—C3B—C4B	−0.7 (4)
O2C—V1—N1A—C7A	34.1 (2)	O1C—C2C—C3C—C4C	−2.6 (5)
V1—O1B—C2B—C1B	−179.51 (16)	C1C—C2C—C3C—C4C	176.4 (3)
V1—O1B—C2B—C3B	0.4 (3)	C2A—C3A—C4A—C5A	1.0 (5)
V1—O1C—C2C—C3C	−9.3 (4)	C2B—C3B—C4B—O2B	0.6 (4)
V1—O1C—C2C—C1C	171.7 (2)	C2B—C3B—C4B—C5B	−179.2 (2)
V1—O2B—C4B—C5B	179.41 (15)	C2C—C3C—C4C—C5C	−174.0 (3)
V1—O2B—C4B—C3B	−0.4 (3)	C2C—C3C—C4C—O2C	5.0 (5)
V1—O2C—C4C—C5C	−176.10 (18)	C3A—C4A—C5A—C6A	−0.2 (5)
V1—O2C—C4C—C3C	4.9 (4)	C4A—C5A—C6A—N2A	−179.6 (3)
V1—N1A—C7A—N2A	−175.13 (16)	C4A—C5A—C6A—C1A	−0.9 (4)
V1—N1A—C1A—C6A	174.97 (15)		

### Hydrogen-bond geometry (Å, °)

**Table:**

*D*—H···*A*	*D*—H	H···*A*	*D*···*A*	*D*—H···*A*
N3A—H3AA···O2B	0.86	2.38	2.972 (3)	127
N3A—H3AA···O2C	0.86	2.47	3.034 (2)	124
C8A—H8AA···N3A	0.96	2.61	2.950 (3)	101
C8A—H8AB···O1C^i^	0.96	2.57	3.146 (3)	119

Symmetry codes: (i) −*x*+3/2, *y*−1/2, −*z*+1/2.
